# Scalable web services for the PSIPRED Protein Analysis Workbench

**DOI:** 10.1093/nar/gkt381

**Published:** 2013-06-08

**Authors:** Daniel W. A. Buchan, Federico Minneci, Tim C. O. Nugent, Kevin Bryson, David T. Jones

**Affiliations:** Department of Computer Science, University College London, Gower Street, London, WC1E 6BT

## Abstract

Here, we present the new UCL Bioinformatics Group’s PSIPRED Protein Analysis Workbench. The Workbench unites all of our previously available analysis methods into a single web-based framework. The new web portal provides a greatly streamlined user interface with a number of new features to allow users to better explore their results. We offer a number of additional services to enable computationally scalable execution of our prediction methods; these include SOAP and XML-RPC web server access and new HADOOP packages. All software and services are available via the UCL Bioinformatics Group website at http://bioinf.cs.ucl.ac.uk/.

## INTRODUCTION

Contemporary computer science is increasingly being defined as the era of ‘Big Data’, as near-unimaginable torrents of data are produced by a great number of human endeavours. This is no less true of Biology. Projects as diverse as ENCODE (1), the 1000 genomes project (2) and SOL-100 (3) amply demonstrate the current and accelerating creation of biological data. All of which is in need of timely, efficient analysis and annotation. The PSIPRED web server aggregates a number of protein annotation tools and provides services or software to allow users to perform truly scalable biological analyses. We have recently rationalized the available methods into two primary homepages—The Protein Analysis Workbench and The Protein Structure Workbench, dealing with protein sequence and protein structure annotation, respectively. We have recently updated a number of the underlying analysis methods and also now provide programmatic access to the web server via SOAP or XML-RPC. As always, we offer the majority of the analysis methods as software downloads, and in addition to this, we now provide HADOOP (4) packages for our most commonly used methods.

## ALGORITHMS AND SOFTWARE

The PSIPRED workbench makes available the following sequence and structure annotation methods: PSIPRED, GenTHREADER, pGenTHREADER, pDomTHREADER, MEMSAT-SVM/MEMSAT3, MEMPACK, BioSerf, MetSite, HSPred, DISOPRED2, DomPred and FFPred. Here, we give a brief overview of the new or updated algorithms available via the PSIPRED workbench; for those algorithms that have not seen major updates, we refer the reader to our previous 2010 Web Server article (5) or seminal publications for particular methods (6).

### BioSerf v2.0

**Figure 1.**
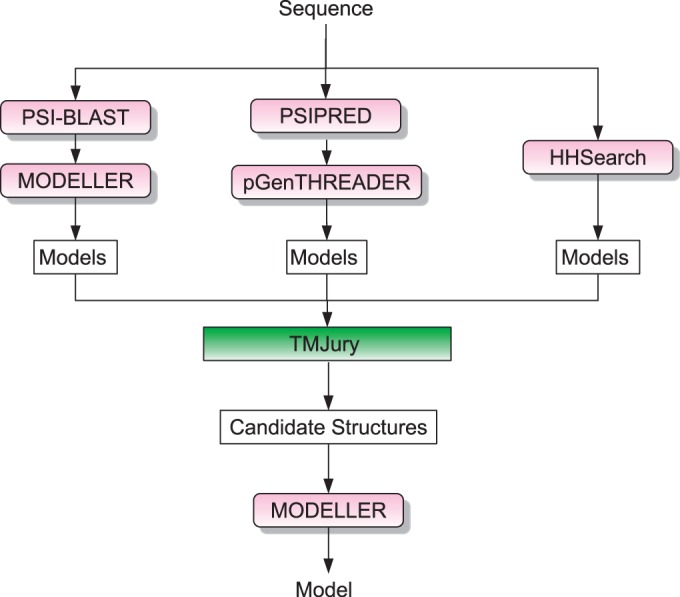
Flowchart of the BioSerf2 automated homology modelling protocol. Incoming query sequences are independently matched to PDB chains using PSIBLAST, pGenTHREADER and HH. The three sets of models produced are then compared by the TMJury process, which produces up to 10 candidate homologous structures. These structures and their alignments to the query sequence are used as input for MODELLER to produce a single final structure.

BioSerf is a fully automated homology modelling protocol, which performed well in the 2008, 2010 and 2012 CASP (7,8) experiments. The BioSerf method has been substantially changed in the last year and no longer makes use of the FRAGFOLD (9) *de novo* modelling software. BioSerf 2 now begins by simultaneously running PSIBLAST (10) against the FASTA PDB (11), running HHSearch (12) against the PDB and also running pGenTHREADER (13). For each method, a list of the best scoring PDB chains is compiled, and a homology model is built for each. pGenTHREADER and HHSearch natively output models, whereas MODELLER (14) is used to build models against the PSIBLAST results. All of the models are then jointly pooled and assessed by calculating the TM-SCORE (15) in an all-against-all manner. This matrix is used to cluster the models into similar sets, and the 10 largest and tightest scoring clusters are then selected, and a PDB model is selected as the cluster representative. The alignments for the top 10 representative models are retrieved from the initial PSIBLAST, HHSearch and pGenTHREADER runs and are then used as input for a final MODELLER job to produce the prediction model. Users of the PSIPRED web server will need a valid MODELLER key to access this prediction method. Figure 1 presents an overview of the process.

### FFPred v2.0

FFPred (16) performs feature-based function prediction and assigns Gene Ontology (GO) classes to query sequences using support vector machines (SVMs), representing each GO class. The method has been extensively updated and re-trained making use of updated versions of all external feature prediction software (manuscript accepted).

Analysis proceeds by annotating a query sequence with a wide range of features, such as secondary structure elements or disordered regions; this information is exploited by each SVM. SVM outputs are converted into posterior probabilities associated to the corresponding annotations using a standard method (17), and they are considered positive predictions whenever the probability is >0.5. The size of the FFPred vocabulary has now increased to include 442 GO classes, 96 from the molecular function domain and 346 in the biological process domain. Also, each GO prediction is assigned a reliability label of either ‘high’ or ‘low’ according to the classifier’s performance in terms of Matthew’s correlation coefficient, sensitivity, specificity and prediction; the 200 best-performing SVMs are assigned ‘high’ reliability, whereas the remainder are assigned ‘low’.

The main output of FFPred consists of a tabulated list of the predicted GO classes providing the GO class code, GO description, GO ontology domain, the posterior probability of the prediction and the reliability level of the SVM. All predictions of GO classes with a ‘high’ reliability level are listed first, whereas predictions of GO classes with ‘low’ reliability are listed against a red background. A summary of the features predicted for the query sequence, which were used for the SVM analysis, is also made available. Finally, users can download two files, one containing the final list of positive predictions, and another one including the posterior probabilities for all of the 442 GO classes.

### MEMSAT-SVM

The support vector machine-based transmembrane topology predictor MEMSAT-SVM (18) has been updated and is now capable of automatically identifying pore-lining regions in transmembrane proteins from sequence information alone. An SVM classifier was trained using sequence profile data by labelling pore-lining residues in crystal structures using geometric criteria and is able to predict the likelihood of a transmembrane helix being involved in pore formation. Results from testing this approach under stringent cross-validation indicate that prediction accuracy of 72% is possible, whereas a support vector regression model is able to predict the pore stoichiometry, i.e. the number of subunits required to form the pore in multimeric proteins, with 62% accuracy. This method provides a way to characterize pores in transmembrane proteins in the absence of crystal structures (19).

### HSPred

HSPred (20) attempts to predict those residues in a protein–protein interface, which make the greatest contribution to the free energy of binding. Protein–protein interactions typically depend on just a handful of energetically critical ‘Hot Spot’ residues. HSPred is an SVM-based method, which attempts to identify such residues. The method takes each interface residue in turn, substitutes it for alanine and then predicts the change in the free energy of binding at the interface. These changes in free energy are then assessed by one of three SVM models; one trained against arginine to alanine mutations, one against glutamic acid to alanine mutations and one for all other substitutions. In predicting ‘Hot Spot’ residues, HSPred achieves a precision and recall of 61 and 69%, respectively.

### HADOOP packages

We regularly receive requests from users wishing to perform large-scale protein annotation projects with one or more of our analysis methods. Because of limited computation resources, we have previously had to direct users to the software downloads for each of our methods. Today accessible and affordable cloud computing is readily available from a large number of vendors. To facilitate very high-throughput protein annotation, we have recently developed a number of HADOOP (4) packages for running our most requested methods, pGenTHREADER, PSIPRED and DISOPRED in a high-throughput manner. These packages allow users to deploy these three methods in a fully scalable fashion on the cloud service of their choice. In the future, we will provide our other analysis methods as and when they are requested. The packages have been tested on the Amazon EC2 service (21) and are available alongside our software downloads via http://bioinf.cs.ucl.ac.uk/software_downloads/.

## WEB SERVER UPDATES

### Online workbench

**Figure 2.**
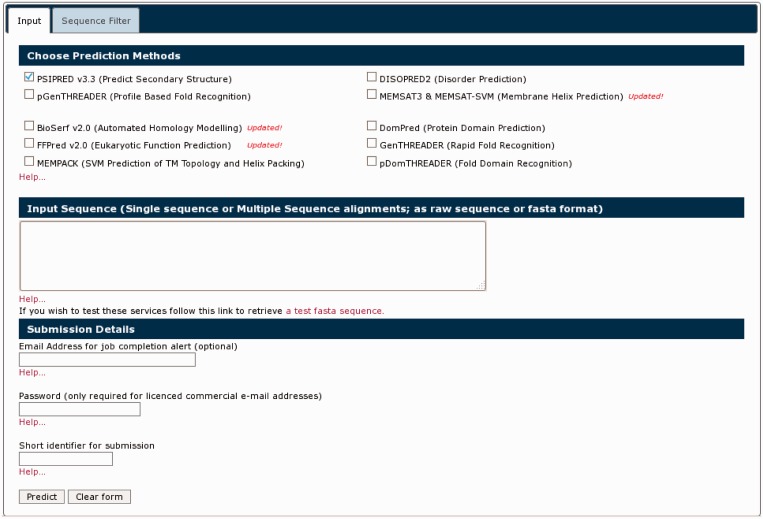
The new front page of the PSIPRED Protein Analysis Workbench. The ‘Choose Prediction Method’ allows users to select any set of available analysis methods. A series of tabs appear along the top bar when users select analysis methods. These additional tabs allow users to select more detailed options.

The previous PSIPRED web server spread many of the available methods across a range of different landing pages with separate input forms. The new PSIPRED Analysis Workbench rationalizes all our methods into one of two main portals: the Protein Analysis Workbench and the Structure Analysis Workbench. These are available at http://bioinf.cs.ucl.ac.uk/psipred/ and http://bioinf.cs.ucl.ac.uk/structure. Users can now select any number of appropriate simultaneous analyses across all the applicable methods and easily explore their results. The input form is now tab based, with expert control options for some analysis methods appearing in pop-up tabs on the page as they are selected (Figure 2).

Many of the sequence analysis jobs critically depend on PSIBLAST as an initial step. This is often the most time consuming process for each job; therefore, the server now attempts to minimize the total number of PSIBLAST jobs performed for a given set of analyses. For instance, PSIPRED, DOMPRED and pGenTHREADER all require PSIBLAST output produced by searching the same sequence database using the same PSIBLAST parameters, any user job that runs these analyses runs only a single PSIBLAST job, greatly reducing the time complex user jobs may take. Similarly, a pGenTHREADER job always begins with a PSIPRED analysis; hence, selecting both tasks runs PSIPRED only once. Alongside this, the server now caches the PSIBLAST output for 6 months; therefore, a sequence that has been seen before will use those results; hence, users need not wait for PSIBLAST to run again to perform the analysis. A typical single analysis now takes between 20 and 40 min, should users select every sequence analysis method, then jobs may take as long as 6 h.

**Figure 3.**
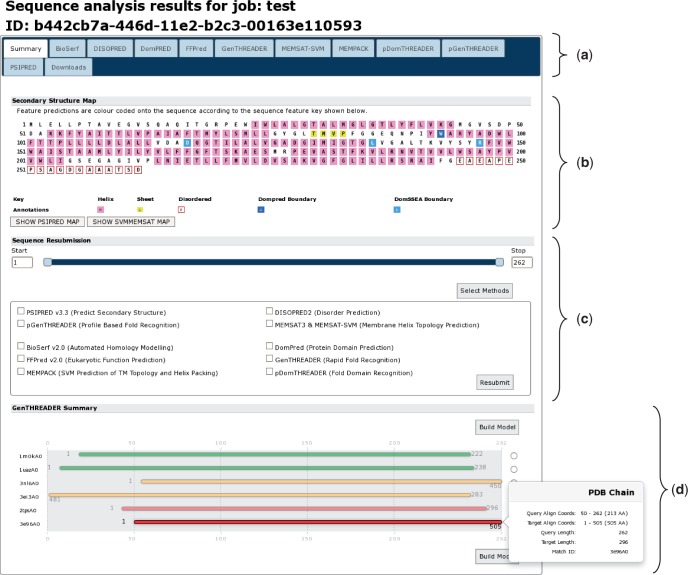
A typical results page where a user has selected all the available analysis methods. The image shows the analysis summary front page of the results. (**a**) Tab bar: this region contains a range of tabs the users can select to explore the detailed results from each analysis method. (**b**) Secondary structure map: this area of the page lays out the query sequence and colours residues as per the annotations made by each analysis methods. If users have selected a MEMSAT-SVM prediction, they can use the buttons provided to toggle between the different sets of sequence annotations. Here, α-helical residues are in pink, β-strand residues are in yellow and putative domain boundaries are indicated in blue. (**c**) Sequence resubmission widget: this region contains a cartoon selector that represents the query sequence. Users can use the sliders to select any sub-sequence of their query sequence and then select further analyses to perform on just the selected sub-sequence. (**d**) GenTHREADER summary: if a GenTHREADER analysis was calculated, the final region presents several schematic cartoons of each GenTHREADER alignment. Hits are presented as bars coloured as per the GenTHEADER confidence scores, green for greatest confidence, orange of moderate confidence and red for lowest confidence. If the user ‘mouses over’ the bars, a pop-up presents more detailed information about the alignment region.

The greatest changes to the user experience can be seen in the results pages (Figure 3). As with the new front page of the server, the results page is now tab based. The first tab and likely the most informative is the sequence summary tab (Figure 3a). This principally presents an annotated schematic of the query sequence, titled ‘Secondary Structure Map’. Each residue is coloured, as per the key, given the annotations determined by the analysis methods, which were run (Figure 3b). If users run a MEMSAT-SVM analysis, they can toggle between the MEMSAT-SVM and other annotations with the buttons provided. Rolling over each residue presents a pop-up with the exact sequence position, the residue three-letter code and details of the predicted residue annotation at that sequence position. Below the secondary structure map is the sequence resubmission widget, this is a cartoon of the query sequence presented as a line with two sliders (Figure 3c). The sliders allow the user to select any arbitrary sub-sequence of their initial query sequence, alternatively residue coordinates can be inputted into the left or right boxes. This selected subsequence can then be resubmitted to the server for further analysis by any of the available methods. To do this, users click the ‘Select Methods’ button, select one or more new analysis methods and then press the submit button.

If users select one of the GenTHREADER, pGenTHREADER or pDomTHREADER methods, the summary page will also display a cartoon of the PDB chain alignments that these methods generate (Figure 3d). The alignments are coloured as per the GenTHREADER confidence scoring boundaries. The aligned PDB chain is noted on the left, and ‘mousing over’ each alignment cartoon presents a pop-up dialogue providing further detailed information about the alignment. To the right of each alignment, users can select a chain and have a simple homology model built using MODELLER (if they have previously provided a valid MODELLER key).

Further tabs, one of each analysis method, allow users to explore the detailed outputs of each analysis method. The final tab allows users to download any output files that were generated. We expect these improvements to our web server’s presentation will better help researchers easily assess their results.

### SOAP/XML-RPC access

Since 2011, the UCL Bioinformatics web server has supported automated, programmatic access to all the prediction methods via both SOAP and XML-RPC. As with regular web server access, users are limited to 20 concurrent jobs. Given the average run time for an analysis, this caps through-put per user to ∼1000 jobs per day. This makes our SOAP service suitable for regularly scheduled low throughput analyses, such as meta-servers, or one-time medium-throughput analyses, such as bacterial proteome annotation.

Full details and instructions can be found at http://bioinf.cs.ucl.ac.uk/web_servers/web_services/. This includes details of all the mandatory submission data for each analysis type and example scripts for submitting jobs and monitoring their progress.

## USE CASE

With the ability to re-submit sub-sequences, users can easily explore their prediction results, queue new predictions and chain together arbitrarily long series of analyses. Here, we present a simple use case that demonstrates these types of use.

We analysed UniProt sequence A9DA50, available via http://www.uniprot.org/uniprot/A9DA50.fasta (22). This mouse protein is currently named ‘Putative transmembrane protein mV/BamHI’. It is also listed as a ‘Leucine-rich repeat immunoglobulin-like domain, containing nogo receptor-interacting protein 1’. The sequence is 620 residues in length. Given the length of the protein and the details in the UniProt entry, it seems probable that this protein has multiple domains. To investigate the domain architecture of this protein, the sequence was submitted to the PSIPRED Sequence Analysis Workbench selecting both the DomPred and pGenTHREADER analyses, to find potential domain boundaries or regions with statistically significant PDB chain matches. Users should note that pGenTHREADER analysis additionally runs a PSIPRED secondary structure prediction. All additional settings were left on their defaults.

**Figure 4.**
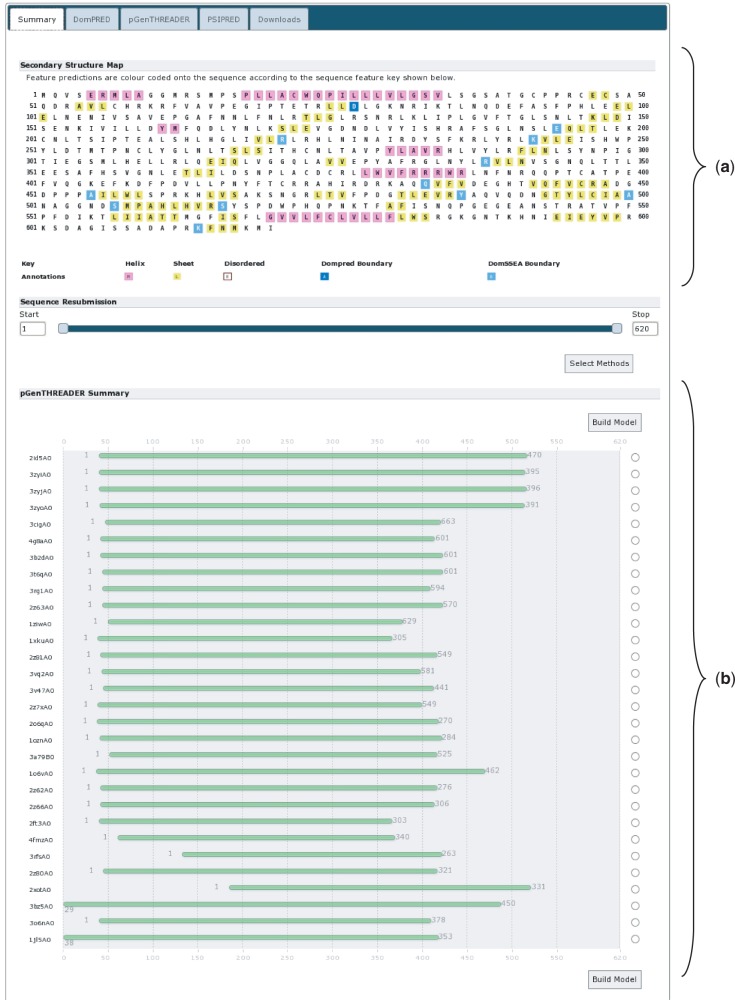
The results summary for UniProt sequence A9DA50. (**a**) The secondary structure map showing the PSIPRED secondary structure predictions. α-Helical residues are in pink, β-strand residues are in yellow and putative domain boundaries are indicated in blue. (**b**) The pGenTHREADER alignments summary. Alignments in green have high-statistical confidence.

The report returned requires some analysis (Figure 4). At the top of the page, the ‘Secondary Structure Map’ (Figure 4a) summarizes the PSIPRED and DomPred output. This indicates an intriguing and seemingly sparse arrangement of secondary structure elements; a recurring and periodic pattern of β-strand elements can be seen beginning at residue 75 (leucine) and running through to residue 365 (isoleucine). This may indicate a structural domain with a highly regular structure in this region. It may also indicate that the sequence begins with a short, 74 residue, N-terminal leading domain and ends with a longer 255 residue C-terminal domain both of which flank the putative periodic β-sheet domain. The DomPred analysis indicates a number of putative domain boundaries. Of particular interest is the first boundary at asparagine 77, this may well correlate to the C-terminal end of a short leading N-terminal domain. For a possible C-terminal domain starting from around residue 365, there are no predictions for domain boundaries that correlate with this location; the closest candidates are at residues 338 or 433.

Looking further down the summary page, the pGenTHREADER alignment cartoons indicate where the pGenTHREADER PDB chain hits are aligned to the query sequence (Figure 4b). The green colouring indicates that these are all hits with strong statistical support. Examining these alignments; there is a clear ‘consensus’ region where several PDB chains have been aligned, running from approximately residue 40 up to at least residue 366 and possibly as far as residue 420. Combined with the DomPred prediction, this may indicate that this is a three domain protein, with a third domain beginning at residue 365 or shortly thereafter.

Taking these observations together, the protein may start with a short N-terminal domain of 40–75 residues and end with a larger C-terminal domain of ∼200–255 residues.

**Figure 5.**
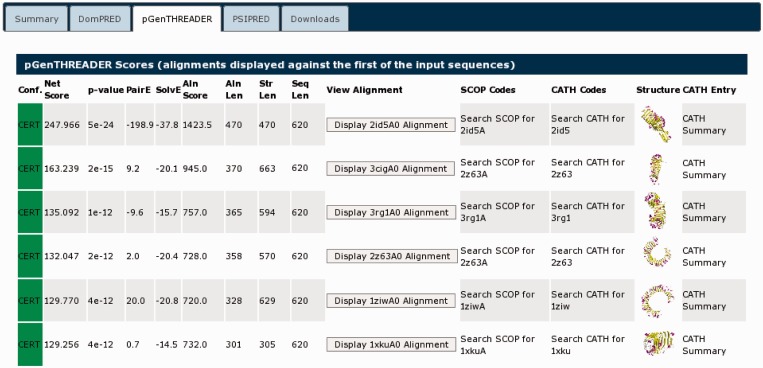
The detailed pGenTHREADER results table for UniProt sequence A9DA50. The matched structures can all be seen to be similar all β-sheet structures. These are listed in CATH and PDBSum as either leucine-rich repeat domains or Tol-like receptors. Those chains fully classified in CATH list this domain in the 3.80.10-fold family.

Selecting the pGenTHREADER tab (Figure 5) displays the detailed pGenTHREADER results. This lists every PDB chain, which matched the query sequence. It can be seen that the structural hits found are β-sheet proteins, somewhat unsurprising as the pGenTHREADER analysis integrates the secondary structure prediction seen under the summary tab. Clicking the thumbnail picture takes the user to PDBSum (23), where a detailed summary of each PDB chain can be found. All the structures listed are identified as either leucine-rich repeats or Tol-like receptors, which is also a known leucine-rich repeat domain. As previously noted from the UniProt entry, this protein may contain a nogo receptor domain (involved in the negative regulation of neuron growth). As the Tol-like receptors are homologues of the nogo receptor domain, all of the matched structures are compatible with the known domain structure of 1ozn, indicating that the PSIPRED Workbench results are in agreement with the UniProt description.

**Figure 6.**
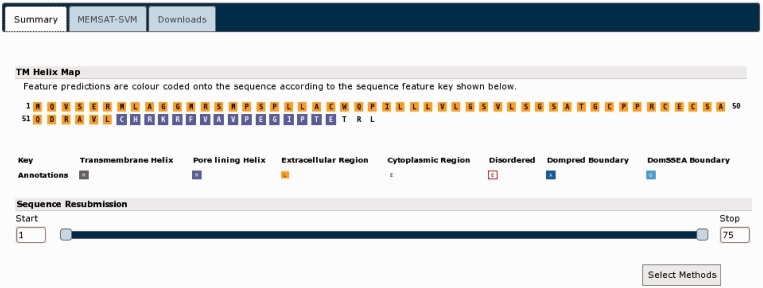
The MEMSAT-SVM results for the N-terminal region of UniProt sequence A9DA50. MEMSAT-SVM predicts a single membrane spanning helix (in purple) in this region.

Returning to the summary page (Figure 4), we can now submit further analyses. The nogo receptor (NRG1) is known to be membrane anchored possessing a short membrane-associated domain. In light of this information, we used the ‘Sequence Resubmission’ tool to select the first 75 residues (as per the initial DomPred prediction), then clicking the ‘Select Methods’ button, this segment was submitted for further analysis with MEMSAT-SVM (Figure 6). Additionally, given evidence from both the alignment consensus seen within the pGenTHREADER cartoon and the DomPred prediction, a C-terminal region between residue 433 and the end of the sequence was resubmitted for a pDomTHREADER analysis in the hope of identifying a CATH (24) structural domain, which may better represent this trailing C-terminal region.

**Figure 7.**
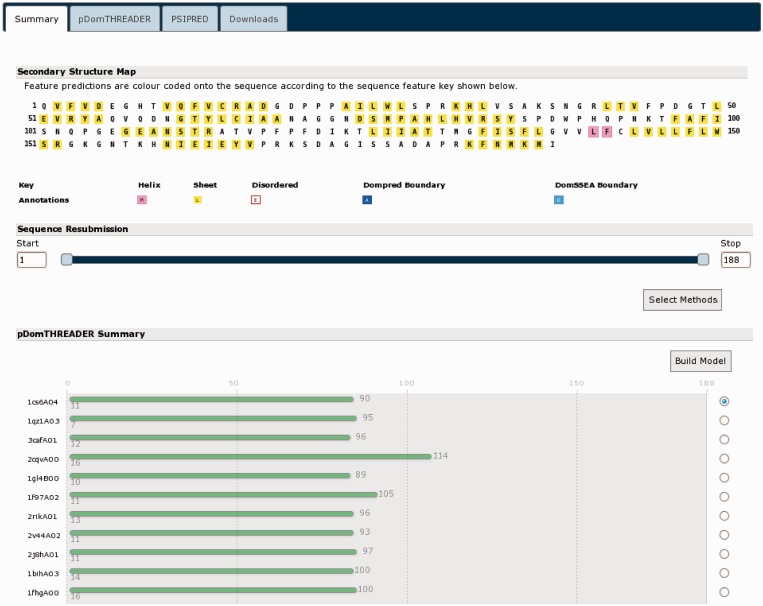
The results summary for the C-terminal region of UniProt sequence A9DA50. The secondary structure and pDomTHREADER results indicate that this region likely has a leading IG-like β-sheet domain.

The MEMSAT-SVM results (Figure 6) indicate that the leading N-terminal domain likely has only a single helix. This is common in membrane-anchored proteins and in keeping with this protein being a homologue of NRG1. The results of the pDomTHREADER analysis (Figure 7) are somewhat less definitive. A very large number of hits are identified (only a selection of the best scoring hits can be shown in Figure 6), but few of the hits cover the length of this sub-domain. The great majority of these alignments cover only the leading 100 residues, which may indicate the C-terminal region possess at least two domains. The initial well-aligned region, given the pDomTHREADER hits, seems to be an all β-sheet domain with an immunoglobulin-like fold. This is followed by an unaligned region representing a yet to be unidentified domain. It would be possible to re-submit this smaller unknown portion for further analysis, but as pDomTHREADER indicates no hits in this region, it is unlikely we could find any further structural domain hits.

To conclude, we are now able to build a good predictive map of the possible domain structure of this protein. The protein starts with a leading N-terminal membrane anchoring domain with a single membrane spanning helix, followed by a longer periodic β-sheet domain homologous to the NRG1 receptor with a structure compatible with PDB structure 1ozn, this is followed by an immunoglobulin domain with the sequence terminating in a final uncharacterized region.

**Figure 8.**
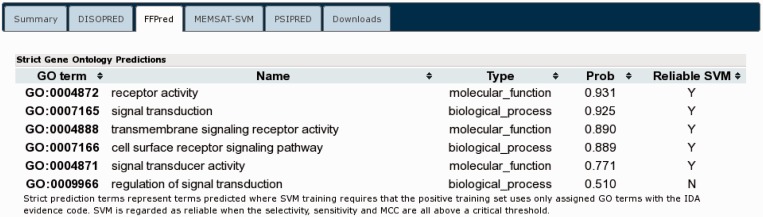
The FFPRED output for UniProt sequence A9DA50 indicating the best matched GO terms. Only the first 10 terms from the list are shown.

These annotations are in good agreement with what little data were available for this sequence at UniProt while additionally adding considerable structural and domain level annotation. Finally, the complete sequence was submitted to FFPred for function prediction (Figure 8). The prediction indicates the sequence is annotated with several functions that agree with our predicted annotation, such as receptor activity (GO:0004872), signal transduction activity (GO:004871) and transmembrane signalling receptor activity (GO:0004888) alongside some related functions. Again this is in good agreement with the annotations already assigned.

## DISCUSSION

The PSIPRED Protein Analysis Workbench offers a great number of accurate protein prediction methods and software. For individual, one-time or bespoke analyses of arbitrary complexity, the software can be accessed and used via the web at http://bioinf.cs.ucl.ac.uk/web_servers/. Additionally, most of the software can also be downloaded for local use (http://bioinf.cs.ucl.ac.uk/software_downloads/).

To enable medium-scale analyses or those computational biologists with limited access to local compute resources, users can now also access all of our web-based services over SOAP/XML-RPC (http://bioinf.cs.ucl.ac.uk/web_servers/web_services/). Finally, for truly massive protein annotation projects, we now offer HADOOP packages for a selection of our methods. These can be deployed on large computer clusters or on any number of commercial cloud compute services (http://bioinf.cs.ucl.ac.uk/software_downloads/). Easy access to this kind of tiered scalable analysis, which can address a variety of user needs, will be essential for the future of computational biology as we enter the post-genomic era.

## FUNDING

Funding for open access charge: Biotechnology and Biological Sciences Research Council (BBSRC), UK.

*Conflict of interest statement.* None declared.
